# Expression of Concern: Validation of a popular consumer-grade cuffless blood pressure device for continuous 24 h monitoring

**DOI:** 10.1093/ehjdh/ztaf134

**Published:** 2025-11-20

This is an expression of concern regarding: Bhavini J Bhatt, Haashim Mohammad Amir, Siana Jones, Alexandra Jamieson, Nishi Chaturvedi, Alun Hughes, Michele Orini, Validation of a popular consumer-grade cuffless blood pressure device for continuous 24 h monitoring, *European Heart Journal - Digital Health*, Volume 6, Issue 4, July 2025, Pages 704-712, https://doi.org/10.1093/ehjdh/ztaf044

In June 2025, the manufacturer of the wearable blood pressure monitor (W-BPM) notified the Journal of concerns regarding the data presented in the article. The concerns involve the authors’ interpretation of the W-BPM manual, resulting in a use of the device that may impact the data collected. The journal is investigating these concerns in line with COPE guidance and is publishing this Expression of Concern while the outcome of the investigation is pending to advise readers to examine the details of this study with particular care.

